# Diagnosis and management of transthyretin familial amyloid polyneuropathy in Japan: red-flag symptom clusters and treatment algorithm

**DOI:** 10.1186/s13023-017-0726-x

**Published:** 2018-01-17

**Authors:** Yoshiki Sekijima, Mitsuharu Ueda, Haruki Koike, Sonoko Misawa, Tomonori Ishii, Yukio Ando

**Affiliations:** 1 Department of Medicine (Neurology and Rheumatology), Shinshu University School of Medicine, Matsumoto, Japan; 2 Department of Neurology, Graduate School of Medical Sciences, Kumamoto University, 1-1-1 Honjo, Chuo-ku, Kumamoto-shi, Kumamoto, 860-8556 Japan; 3 Department of Neurology, Nagoya University Graduate School of Medicine, Nagoya, Japan; 4 Department of Neurology, Graduate School of Medicine, Chiba University, Chiba, Japan; 5 Pfizer Japan Inc., Tokyo, Japan

**Keywords:** Disease-modifying agent, Tafamidis, Liver transplantation, Hereditary ATTR amyloidosis, Familial amyloid polyneuropathy, Amyloidosis neuropathy, Carpal tunnel syndrome, Cardiomyopathy, Red-flag symptom clusters

## Abstract

Hereditary ATTR (ATTRm) amyloidosis (also called transthyretin-type familial amyloid polyneuropathy [ATTR-FAP]) is an autosomal-dominant, adult-onset, rare systemic disorder predominantly characterized by irreversible, progressive, and persistent peripheral nerve damage. *TTR* gene mutations (e.g. replacement of valine with methionine at position 30 [Val30Met (p.Val50Met)]) lead to destabilization and dissociation of TTR tetramers into variant TTR monomers, which form amyloid fibrils that deposit in peripheral nerves and various organs, giving rise to peripheral and autonomic neuropathy and several non-disease specific symptoms.

Phenotypic and genetic variability and non–disease-specific symptoms often delay diagnosis and lead to misdiagnosis. Red-flag symptom clusters simplify diagnosis globally. However, in Japan, types of TTR variants, age of onset, penetrance, and clinical symptoms of Val30Met are more varied than in other countries. Hence, development of a Japan-specific red-flag symptom cluster is warranted. Presence of progressive peripheral sensory-motor polyneuropathy and ≥1 red-flag sign/symptom (e.g. family history, autonomic dysfunction, cardiac involvement, carpal tunnel syndrome, gastrointestinal disturbances, unexplained weight loss, and immunotherapy resistance) suggests ATTR-FAP. Outside of Japan, pharmacotherapeutic options are first-line therapy. However, because of positive outcomes (better life expectancy and higher survival rates) with living donor transplant in Japan, liver transplantation remains first-line treatment, necessitating a Japan-specific treatment algorithm.

Herein, we present a consolidated review of the ATTR-FAP Val30Met landscape in Japan and summarize findings from a medical advisory board meeting held in Tokyo on 18th August 2016, at which a Japan-specific ATTR-FAP red-flag symptom cluster and treatment algorithm was developed. Beside liver transplantation, a TTR-stabilizing agent (e.g. tafamidis) is a treatment option. Early diagnosis and timely treatment using the Japan-specific red-flag symptom cluster and treatment algorithm might help guide clinicians regarding apt and judicious use of available treatment modalities.

## Background

Transthyretin-type familial amyloid polyneuropathy (ATTR-FAP), or hereditary transthyretin amyloidosis (ATTRm amyloidosis), is an autosomal-dominant, adult-onset, rare systemic disorder predominantly characterized by irreversible, progressive, and persistent peripheral nerve damage [1, 2]. ATTR-FAP can present as a progressive, axonal, sensory autonomic and motor neuropathy, restrictive cardiomyopathy (transthyretin cardiomyopathy), or as a cerebral amyloid angiopathy; however, most cases are classified as neuropathic [1]. In analysis of data from the Ministry of Health, Labour and Welfare, Japan (MHLW), during 2003–2005, 110.8–135.4 cases of familial amyloidosis were found, equating to an estimated prevalence of 0.87–1.1 per 1,000,000 persons; the highest prevalence was in the Nagano prefecture, followed by Kumamoto, and Ishikawa (11.0–15.5, 10.1–10.3, and 3.5–4.2 per 1,000,000 persons, respectively) [3].

TTR—a homotetramer plasma transport protein that carries thyroxine and retinol-binding protein—is produced primarily in the liver but also in the choroid plexus and retinal pigment epithelium, and is secreted into the blood, cerebrospinal fluid, and eye, respectively [1, 4–9].

**Fig. 1:**
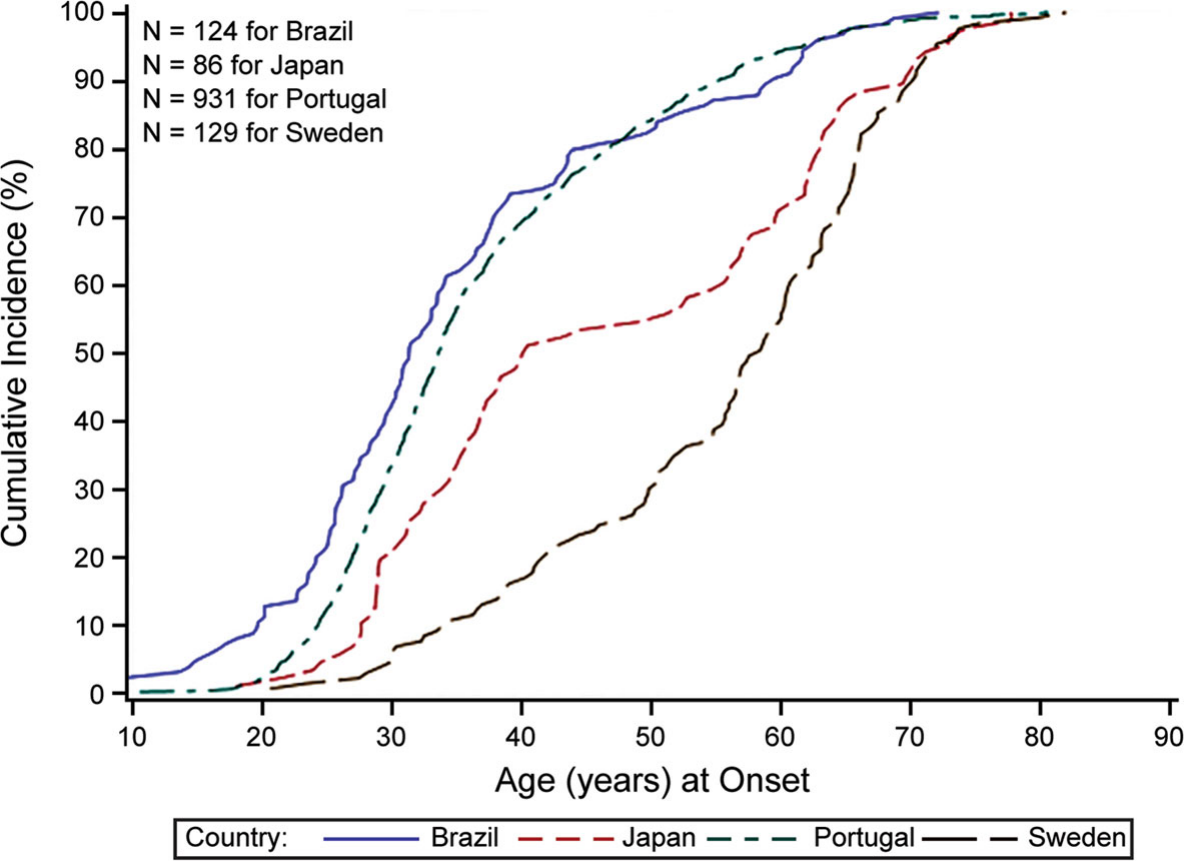
Cumulative onset of symptomatic disease: Val30Met in four countries

There are over 130 different *TTR* gene mutations identified worldwide, of which >40 mutations are linked with ATTR-FAP in Japan (Table 1). However, replacement of valine with methionine at position 30 (ATTR-FAP Val30Met [p.Val50Met]) is the most commonly observed mutation, the only one found in large foci of patients, and is associated primarily with neuropathy [1, 10]. *TTR* gene mutations destabilize TTR, leading to dissociation of tetramers and partial unfolding of resultant monomers. Variant TTR monomers aggregate to form amyloid fibrils [1, 11–13]. Because of diffuse amyloid fibril deposition (e.g. in the extracellular space in peripheral nerves, heart, gastrointestinal tract, kidneys, eyes, meninges, vessels, and connective tissue of the transverse carpal ligament) [14], ATTR-FAP is associated with various symptoms, many of which are non-specific [1, 11, 15]. Disease onset, which occurs between the 10s and 90s, is generally classified as early-onset (<50 years old) and late-onset (≥50 years old) [16, 17]. Within approximately 10 years of disease onset, progressive organ dysfunction and death (due to cardiac dysfunction, infection, or cachexia) occur [1, 18–21]. Several studies have indicated that onset of ATTR-FAP symptoms in Japan is bimodal, with one peak occurring in the 30s to 40s (early-onset) and another distinct peak in the 60s (late-onset) [1, 3, 16, 22–24]. In contrast, other countries have a single peak of symptom onset (e.g. between 25 and 35 years with a mean of 33.5 years in Portugal and in the 50s or 60s in Sweden) [25–27] (Fig. 1; Pfizer Inc., data on file).

**Table 1:** ATTR-FAP linked genetic mutations in Japan

Main symptoms	Genetic mutations
Peripheral neuropathy	Ala25Ser, Val30Leu, Phe33Val, Asp38Ala, Glu42Gly, Phe44Ser, Gly47Arg, Gly47Val, Thr49Ile, Thr49Ala, Ser50Arg, Glu54Lys, Leu55Pro, Glu61Lys, Val71Ala, Ser77Tyr, Ala97Gly, Ala109Ser, Val28Ser, Val28Met, Ala36Pro, Ile84Asn, His88Arg, Ala120Ser
CTS + peripheral neuropathy	Leu58Arg, Tyr69Ile, Ile107Val, Tyr114His, Ala120Ser, Ala120Thr
Cardiac	Asp18Glu, Ala36Asp, Ala45Asp, Ser50Ile, Thr59Arg, Thr60Ala, Glu89Lys, Gln92Lys, Val94Gly, Asp38Ala, Ser50Arg, Val122Ile, Glu89Gln, Pro24Ser, Val30Leu
Leptomeningeal	Ala25Thr, Gly53Glu, Tyr114Cys, Asp18Gly, Tyr69His
Non-pathologic	Arg104His
Compound heterozygosity	Val30Met/Arg104His

*ATTR-FAP* Transthyretin familial amyloid polyneuropathy, *CTS* Carpal tunnel syndrome

In addition to heterogeneity of amyloidogenic *TTR* mutations and variability in the age of onset, phenotypic heterogeneity at various levels makes ATTR-FAP diagnosis challenging. Carriers of the same *TTR* point mutation may exhibit very different clinical manifestations, even among family members [1]. Also, absence of family history in non-endemic areas, and sporadic cases present additional diagnostic challenges [28–31]. As ATTR-FAP is a progressive disease and can cause largely irreversible tissue damage, timely recognition and diagnosis are critical for appropriate treatment and optimal outcomes [32–34]. Unfortunately, diagnosis of ATTR-FAP is often delayed because of phenotypic and genetic variability, varied clinical presentation, and the non-specific nature of most symptoms [1, 19, 30, 31, 35]. Misdiagnosis also may occur for similar reasons (see Common diagnostic pitfalls).

In the past decade, the situation surrounding ATTR-FAP and its treatment has changed dramatically in Japan: owing to heightened awareness of the disease, the number of ATTR-FAP patients in this country has reportedly increased from 110.8–135.4 [3] to approximately 300 (Sekijima et al., unpublished observations). In terms of treatment, liver transplantation (LT) has been the only standard of care since the 1990s [1, 36–41]; in the early 2010s, however, treatment options were widened by the advent of minimally invasive, disease-modifying pharmacotherapy such as TTR tetramer stabilizer [1, 35, 42]. Nevertheless, a number of patients with ATTR-FAP still remain undiagnosed and thus untreated because of diverse clinical presentations and various non-specific symptoms of the disease; especially in Japan, presence of diverse types of patients (e.g. early-onset Val30Met in endemic areas, late-onset Val30Met in non-endemic areas, non-Val30Met variants whose cardinal symptoms are cardiomyopathy, carpal tunnel syndrome, or cerebral amyloid angiopathy) often makes accurate diagnosis difficult [15, 16, 24, 43–45].

In view of the enormous possibility of misdiagnosis or delayed diagnosis, Conceição and colleagues reported red-flag symptom clusters suggestive of ATTR-FAP and treatment algorithms [46]. However, these clusters and algorithms are not necessarily applicable to Japan because situations specific to this country (e.g. presence of diverse types of patients) are not fully reflected. Herein, we provide a consolidated review of the worldwide landscape of ATTR-FAP and our treatment experience in Japanese ATTR-FAP patients to propose revised red-flag symptom clusters and treatment algorithm.

## Methods

The authors (YS, MU, HK, SM, and YA) held a medical advisory board meeting in Tokyo, Japan on 18th August 2016 with the aim to promote early diagnosis and to stipulate consensus on diagnosis and management of ATTR-FAP Val30Met in Japan. The red-flag symptom clusters and treatment algorithm presented in this article are developed as per findings from this medical advisory board meeting.

### Clinical features

ATTR-FAP Val30Met can be endemic (i.e. localized to a small area, with a traceable family history and early-onset of the disease) or non-endemic (i.e. scattered, frequently without family history, and late-onset of the disease) [3, 16, 29–31, 34, 47]. The clinical picture of ATTR-FAP differs between patients from endemic and non-endemic areas [1, 15, 16, 48]. Generally, patients from endemic areas have early-onset disease, while those from non-endemic areas have late-onset disease [15, 16, 20, 44, 49]. In Japan, however, despite an identical *TTR* genotype and generally homogenous ethnic background, two major ATTR-FAP Val30Met phenotypes have been identified: early-onset and endemic (Nagano and Kumamoto), and late-onset and non-endemic [16, 20, 31]. Common clinical features of the early-onset and endemic phenotype in Japan resemble those of Portuguese FAP patients [10, 27, 50, 51], while clinical features of the late-onset phenotype in non-endemic areas are distinct from those phenotypes [16, 20, 31]. Differences in clinical features between early-onset and late-onset disease are presented in Table 2. Early-onset ATTR-FAP generally starts between the late 20s to the early 40s and is characterized by predominant loss of superficial sensation including nociception and thermal sensation (i.e. sensory dissociation), presence of family history, high penetrance rate, severe autonomic dysfunction, and atrioventricular conduction block requiring pacemaker implantation [15, 16, 49, 52–54]. On the other hand, late-onset disease starts after 50 years of age and is characterized by sensorimotor symptoms beginning in the distal lower extremities, initial involvement of both superficial and deep sensation, loss of all sensory modalities rather than sensory dissociation, low penetrance rate, relatively mild autonomic dysfunction, frequent presence of cardiomegaly, and extreme male preponderance [15, 16, 24, 31, 55]. Genetic anticipation is frequently considered a differentiating feature due to its presence in early-onset disease and its absence in late-onset disease [43, 56]. However, the occurrence of anticipation may be overestimated since not all asymptomatic individuals undergo molecular genetic testing, and hence some asymptomatic individuals with the Val30Met variant may not be identified [57].

**Table 2:** Comparison of clinical features between early-onset and late-onset ATTR-FAP

Clinical feature	Early-onset	Late-onset
Age of onset of symptoms	• Late 20s to early 40s [79]	• ≥50 years [16, 20, 24]
Penetrance	• High penetrance rate [16]	• Low penetrance rate [24]
Pattern of neuropathic symptoms	• Loss of superficial sensation, including nociception and thermal sensation (i.e. sensory dissociation) [16]	• Loss of all sensory modalities rather than sensory dissociation. Impaired superficial and deep sensation, neuropathic pain, early distal motor involvement [31, 92]
Family history of ATTR-FAP	• Common [24]	• Frequently absent [24]
Autonomic dysfunction	• Severe, life-threatening autonomic dysfunction [16]	• Relatively mild autonomic symptoms [16]
Other features	• Atrioventricular conduction block requiring pacemaker implantation • Weight loss • Muscle wasting [16]	• Extreme male preponderance • Frequent presence of cardiomegaly [92, 157]

*ATTR-FAP* Transthyretin familial amyloid polyneuropathy

**Fig. 2:**
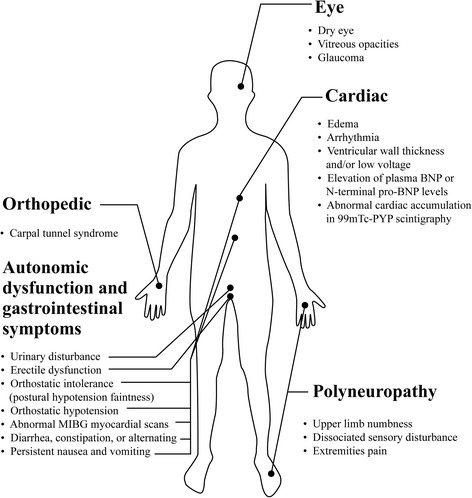
Clinical and paraclinical features of ATTR-FAP. *BNP* brain natriuretic peptide, *Tc-PYP* Tc-pyrophosphate scintigraphy, *MIBG* metaiodobenzylguanidine

The clinical and paraclinical features of ATTR-FAP are presented in Fig. 2. In general, fiber length-dependent peripheral sensory-motor neuropathy is a hallmark feature of ATTR-FAP [1, 15]. A number of previously reported studies have discussed initial symptoms in ATTR-FAP patients in Japan [15, 16, 20, 31, 45, 46, 50]. The initial symptoms in Japanese ATTR-FAP patients are presented in Table 3. Symptoms of ATTR-FAP can be broadly divided into neuropathic symptoms and other systemic symptoms as described below.

**Table 3:** Initial symptoms of ATTR-FAP Val30Met patients in Japan

	Ando Y et al., 2005 [52]	Ikeda S et al., 1987 [53]	Koike H et al., 2002 [16]		Koike H et al., 2012 [20]
		Early onset^a^	Early onset^a^	Late onset^b^	Late onset^b^
Patients, *n*	117	45	82	59	50
Mean ± SD age of onset, years	35.3	33.4^c^, 34.2^d^	31.9 ± 7.6	62.5 ± 6.2	64.5 ± 6.5
Sensory-motor symptoms, *n* (%)					
Sensory disturbances in lower limbs	52 (44.4)	22 (48.9)			
Neuropathic symptoms			47 (57.3)	48 (81.4)	40 (80.0)
Carpal tunnel syndrome					
Muscle weakness in lower limbs	3 (2.6)	3 (6.7)			
Autonomic and GI symptoms, *n* (%)	48 (41.0)	19 (42.2)	39 (48.0)	6 (10.2)	
Autonomic symptoms	10 (8.5)				5 (10.0)
Erectile dysfunction/impotence	5 (4.3)	4 (8.9)			
Orthostatic hypotension/faintness/syncope	5 (4.3)	3 (6.7)			
GI symptoms	38 (32.5)				
Anorexia		2 (4.4)			
Constipation		8 (17.8)			
Diarrhea		2 (4.4)			
Weight loss, *n* (%)			4 (4.9)	0	
Cardiac symptoms, *n* (%)	5 (4.3)		0	3 (5.1)	2 (4.0)
Renal dysfunction, *n* (%)	5 (4.3)				
Ocular symptoms, *n* (%)	4 (3.4)		0	1 (1.7)	3 (6.0)
Bullous formations, *n* (%)		1 (2.2)			

*ATTR-FAP* Transthyretin familial amyloid polyneuropathy, *GI* Gastrointestinal, *SD* Standard deviation*, Val30Met* Replacement of valine with methionine at position 30 in the TTR gene

^a^Age <50 years at symptomatic disease onset

^b^Age ≥50 years at symptomatic disease onset

^c^Men, *n* = 23 (51.1%)

^d^Women, *n* = 22 (48.9%)

#### Neuropathic symptoms

In classical early-onset disease, damage is first observed in distal small myelinated and unmyelinated nerve fibers associated with pain and temperature and manifests as paresthesia, dysesthesia, allodynia, hyperalgesia, or spontaneous pain in the feet [15, 16] and impaired thermal sensitivity with decreased pinprick sensation on clinical examination [1, 15, 16]. Larger myelinated sensory and motor nerve fibers are affected over the following years, impairing light touch, vibration, and position sensation. Further length-dependent progression leads to distal lower limb motor deficit, resulting in walking difficulty and weakness [15]. In late-onset disease, unmyelinated nerve fibers are preserved, and axonal sprouting is observed [15]. Autonomic dysfunction presents as sexual impotence; disturbances of gastrointestinal motility, most commonly diarrhea alternating with constipation but also constipation, diarrhea, nausea, and vomiting; orthostatic hypotension; and neurogenic bladder [16, 20, 58]. These autonomic symptoms are relatively mild in late-onset disease particularly in the early phase of neuropathy [16, 20]. Symptoms of the lower limbs usually precede those of the upper limbs by several years in early-onset disease, while the involvement of the upper and lower limbs may appear simultaneously in late-onset disease [20]. Occasionally carpal tunnel syndrome (CTS) may appear in the patients with non-Val30Met and lead to diagnosis in the progression of systemic neuropathy after carpal tunnel release surgery [1, 59–62].

#### Other systemic symptoms

In addition to nervous tissue, amyloid fibrils may deposit in various organs and tissues resulting in progressive dysfunction [1, 14, 15, 63–66]. Amyloid deposition in the media and adventitia of medium-sized and small arteries, arterioles, and, occasionally, veins of the subarachnoid space, leptomeninges, and cerebral cortex leads to transient focal neurological episodes, cerebral infarction and hemorrhage, hydrocephalus, ataxia, spastic paralysis, convulsion, and dementia [1, 61, 62, 64, 67]. Infiltration of amyloid fibrils in cardiovascular structures such as the conduction system may lead to bundle branch block and, occasionally, atrioventricular and sinoatrial block [15]. Myocardial infiltration may lead to cardiomyopathy, with a hypertrophic phenotype and restrictive pathophysiology [1, 68]. Deposition of amyloid fibrils in the eye may cause ocular manifestations such as abnormal conjunctival vessels, keratoconjunctivitis sicca, pupillary abnormality, vitreous opacity, and glaucoma [69]. Amyloid fibril deposition in the kidney might lead to micro-albuminuria, which often precedes subjective symptoms of ATTR-FAP. Renal involvement, including nephritic syndrome and progressive renal failure, occurs in about one-third of patients in Portugal [70]; however, severe renal dysfunction rarely occurs in Japanese ATTR-FAP patients. Further, as the kidney is the major site of erythropoietin production, anemia might develop because of significantly lower serum erythropoietin levels [71].

#### Non-specific symptoms

Weight loss, muscle wasting and atrophy, hoarseness, coldness, decreased skin temperature, dyscoria, dysesthesia, dissociated anesthesia, arrhythmia, edema, burning, and Charcot’s joint also may be present in patients with ATTR-FAP [1, 16].

### Japan-specific red flag symptom clusters

**Fig. 3:**
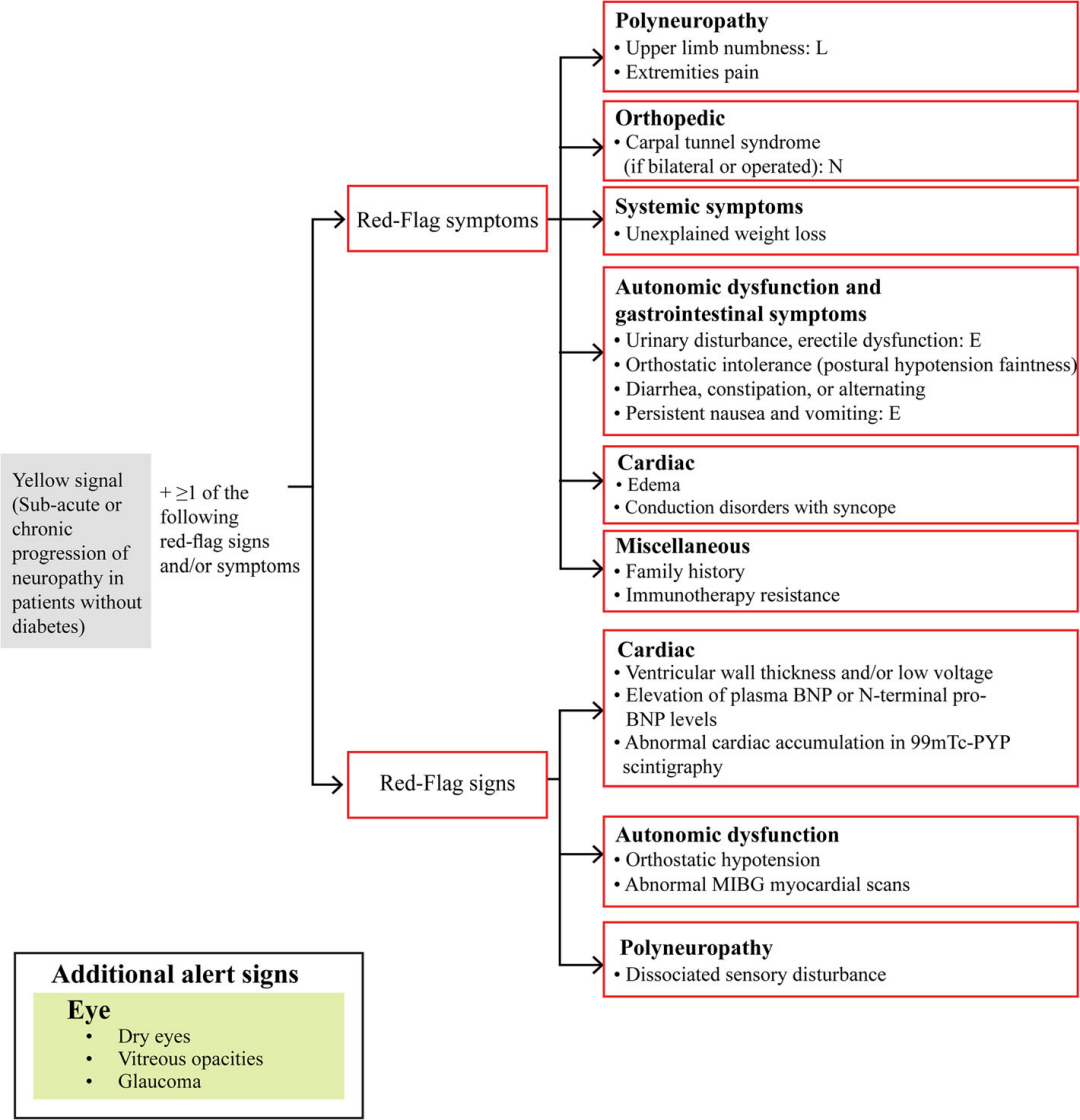
Red-flag symptom clusters specific to ATTR-FAP Val30Met in Japan. *E* early-onset Val30Met, *L* late-onset Val30Met, *N* non-Val30Met, *BNP* brain natriuretic peptide, *Tc-PYP* Tc-pyrophosphate scintigraphy, *MIBG* metaiodobenzylguanidine

Heritability and multisystem involvement are characteristic features of ATTR-FAP [46]. Red-flag symptom clusters suggestive of ATTR-FAP reported by Conceição and colleagues included: family history; early autonomic dysfunction; gastrointestinal complaints; unexplained weight loss; cardiac hypertrophy, arrhythmias, ventricular blocks, or cardiomyopathy; bilateral CTS; renal abnormalities; and vitreous opacities [46]. In the light of published literature and the medical advisory board’s expert opinion, red-flag symptom clusters suggesting ATTR-FAP in Japan are reviewed and presented below (Fig. 3). The presence of progressive peripheral sensory-motor polyneuropathy and ≥1 of the following red-flag signs and/or symptoms is suggestive of ATTR-FAP.

#### Gastrointestinal symptoms

Gastrointestinal symptoms such as nausea, early satiety, recurrent vomiting, watery diarrhea, severe constipation, and/or alternating diarrhea and constipation that occur as manifestations of autonomic neuropathy are documented early on in ATTR-FAP [1] and are the initial symptoms in nearly half of early-onset cases in endemic areas [16, 72, 73]. Patients from non-endemic areas mainly present with lower gastrointestinal tract symptoms such as diarrhea and/or constipation [20]. Notably, Japanese patients have an earlier onset of gastrointestinal disturbances than Swedish patients [74], making it an important red-flag symptom in Japanese patients.

#### Carpal tunnel syndrome

CTS is an early but non-specific orthopedic manifestation of ATTR-FAP. Often, ATTR-FAP patients are initially misdiagnosed with idiopathic CTS, and progressive symptoms or lack of improvement after release surgery often leads to the correct diagnosis. Therefore, CTS without obvious cause, particularly bilateral CTS that requires surgical release, should raise suspicion of ATTR-FAP [1]. In a retrospective, observational study involving 76 Italian ATTR-FAP patients, CTS was an inaugural symptom in 33% patients, with no other clinical manifestations for a mean period of 4.6–5.6 years [75]. Likewise, in a study involving 31 Japanese patients diagnosed with systemic wild-type transthyretin amyloidosis at Shinshu University Hospital, CTS was the most common initial symptom, indicating that careful examination of patients with CTS may lead to earlier diagnosis [76].

#### Unexplained weight loss

Unintentional weight loss is frequently observed in ATTR-FAP patients because of gastrointestinal disturbances [1]. Cachexia is a major cause of death in early-onset ATTR-FAP Val30Met patients from endemic foci in Japan and Portugal [50, 73, 77].

#### Autonomic dysfunction

Although sensory and motor manifestations are generally presenting symptoms, autonomic dysfunction can be the first clinical presentation in early-onset cases [49]. In a nationwide survey conducted by the Study Group for Hereditary Neuropathy (under the auspices of the MHLW), autonomic dysfunction was the initial complaint in 48% of early-onset and 10% of late-onset cases [16]. Autonomic symptoms in late-onset ATTR-FAP are generally mild in the early phase of the disease [31]. However, autonomic dysfunction usually becomes apparent in the later phase of the disease, even in late-onset cases [20]. Further, as inadequate attention of neurologists to autonomic symptoms is a major diagnostic pitfall in ATTR-FAP, special attention must be paid to patients with concurrent autonomic dysfunction, CTS, and cardiac involvement [1, 19, 31].

#### Cardiac involvement

Approximately 50% of patients with ATTR-FAP experience cardiac disease [1], and cardiac dysfunction is the major cause of death, particularly among patients from non-endemic areas [20, 78]. Although signs and symptoms of cardiac disease generally appear in the later phase of ATTR-FAP, early assessments might reveal cardiac involvement [20]. Detection of subclinical cardiac involvement (e.g. cardiomegaly on chest X-ray, and thickening of the interventricular septum and granular sparkling on echocardiography [31]) may help diagnose late-onset ATTR-FAP Val30Met in patients without a family history of the disease [79]. Furthermore, detection of uptake of technetium-99m-pyrophosphate with cardiac scintigraphy helps early diagnosis of TTR-cardiac amyloidosis with high sensitivity and specificity [80, 81].

#### Family history

In the aforementioned nationwide survey conducted in Japan, family history of ATTR-FAP Val30Met was found in 94% of early-onset and 48% of late-onset cases [16]. Despite a lower incidence of family history among patients with late-onset disease and those in non-endemic areas [16, 24, 79], red-flag symptom clusters should raise suspicion of ATTR-FAP, particularly in those with a family history. Further, experienced neurologists in endemic areas might possibly diagnose ATTR-FAP solely based on family history and clinical features [1, 46].

#### Immunotherapy resistance

Failure to respond to immunomodulatory treatment helps to differentiate ATTR-FAP from chronic inflammatory demyelinating polyneuropathy (CIDP), which is the most common misdiagnosis if associated with steady progression of the neuropathy, an axonal pattern, and autonomic dysfunction [31, 46].

The knowledge and awareness of the above red-flag symptom cluster among physicians in Japan may provide practical direction and promote early identification and diagnosis of the disease in this country.

### Diagnosis

**Fig. 4:**
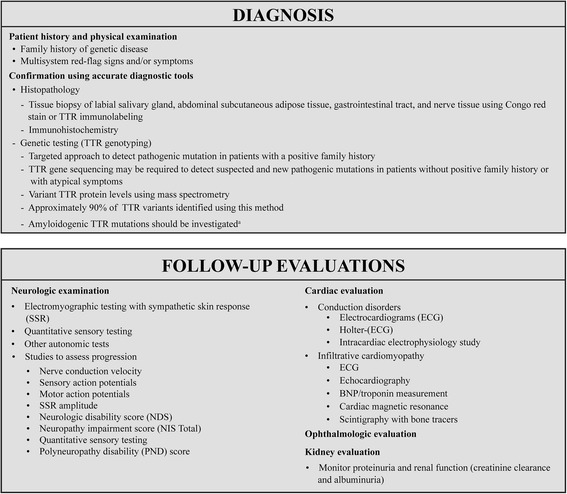
Diagnostic tools and follow-up evaluations for ATTR-FAP. *BNP* brain natriuretic peptide, *ECG* electrocardiogram, *NDS* neurologic disability score, *NIS* neuropathy impairment score, *PND* polyneuropathy disability, *SSR* sympathetic skin response, *TTR* transthyretin. ^a^Rowczenio DM, et al. 2014 [94]

Diagnosis of ATTR-FAP involves two primary steps [82]:

1. Patient history and physical examination, which may raise clinical suspicion and permit a tentative diagnosis of ATTR-FAP
2. Confirmation using accurate diagnostic tools, including histopathology and genetic analysis (Fig. 4)

#### Patient history and physical examination

A thorough clinical history of the patient should be taken to identify the presence of family history and the multisystem red-flag signs and/or symptoms [1, 82]. In the absence of a family history of amyloidosis, the diagnosis of ATTR-FAP should be considered in patients with a progressive, length-dependent, axonal polyneuropathy predominantly affecting temperature and pain sensation [1] (Fig. 4). After diagnosis, the modified body mass index (mBMI) as a measure of nutritional status is helpful to monitor progression or prognosis of ATTR-FAP [1, 83].

#### Histopathology

Tissue biopsy: Demonstrating amyloid deposits via tissue biopsy is essential to confirm an ATTR-FAP diagnosis, especially in patients without a family history [1, 84, 85]. Tissue biopsy using Congo red stain [85] directly reveals amyloid deposits in affected tissues, including the labial salivary gland and abdominal subcutaneous adipose tissue, gastrointestinal tract, nerve tissue, and other organs with evidence of involvement [18, 31, 86–90]. TTR immunolabeling of amyloid deposits helps identify TTR amyloidosis [82] but does not aid differentiation between wild-type ATTR (ATTRwt) and mutant ATTR (ATTRm). Further, in the presence of typical signs and symptoms, negative biopsy results do not rule out ATTR-FAP [1] (Fig. 4).

#### Genetic testing

In patients with suspected ATTR-FAP, TTR genotyping should be performed to document the specific pathogenic *TTR* mutations; genotyping is the most reliable diagnostic approach, and absence of a pathogenic mutation excludes diagnosis of ATTR-FAP [1, 82]. *TTR* genopositivity should be established by DNA analysis in all suspected cases [1, 30, 91–93]. In patients having family history with previous diagnosis, a targeted approach can be used to detect the pathogenic mutation. In the absence of family history and in patients with atypical symptoms, *TTR* gene sequencing may be required to detect suspected and new pathogenic mutations [35, 57]. Further, an online registry will prove useful to investigate amyloidogenic *TTR* mutations [94] (Fig. 4).

#### Serum variant TTR protein

TTR protein normally circulates in serum as a soluble protein with a tetrameric structure. The normal serum TTR concentration is 0.20 to 0.40 mg/mL (20 to 40 mg/dL) [57, 95, 96]. After immunoprecipitation with anti-TTR antibody and dissociation of the tetrameric structure of TTR (into pro-amyloidogenic monomers), serum variant TTR protein can be detected by mass spectrometry [97–100]. Approximately 90% of TTR variants are identified by this method and they exhibit the mass shift predicted by the one amino acid substitution of the variant TTR [57, 97, 101] (Fig. 4).

#### Neurologic examination

On the basis of presenting signs and symptoms, patients should undergo a complete neurological examination to identify, characterize, and measure the severity of neuropathic abnormalities involving small and large nerve fibers [1, 82]. Scores used to assess neuropathy, and local variants and scales that quantify neurologic function in patients with diabetic polyneuropathy but are useful for patients with ATTR-FAP, should also be used to assess neuropathic symptoms [1]. Likewise, nerve conduction velocity, sensory action potentials, and other tests for characterizing small-fiber (coolness and heat detection) and large-fiber (vibratory detection) peripheral sensory thresholds should be used to evaluate ATTR-FAP progression [1] (Fig. 4).

Following diagnosis and assessment of neurological symptoms, systemic extension of the disease should be determined via assessment of heart, eyes, kidney, etc. [1, 82].

#### Cardiac evaluation

Cardiac investigations should be conducted to detect infiltrative cardiomyopathy and serious conduction disorders carrying risk of sudden death [1] (Fig. 4).

#### Ophthalmologic evaluation

Ophthalmological assessment is necessary to identify possible ocular manifestations such as keratoconjunctivitis sicca, secondary glaucoma, vitreous opacities, or pupillary abnormalities [69, 102] (Fig. 4).

#### Kidney evaluation

In view of possible microalbuminuria, and/or mild azotemias and subsequent renal failure, monitoring for proteinuria and abnormal renal function (creatinine clearance and albuminuria) parameters is recommended in ATTR-FAP patients [70, 82, 103].

### Common diagnostic pitfalls

Though length-dependent sensory-motor polyneuropathy is a hallmark feature of ATTR-FAP, it is not distinctive of this condition and can be present in more prevalent neurological conditions, potentially leading to misdiagnoses [82, 92].

#### CIDP

CIDP, which is characterized by a demyelinating sensory-motor neuropathy, is the most common neuropathic misdiagnosis for sporadic ATTR-FAP. In one study, 53% of 15 Japanese patients with sporadic ATTR-FAP Val30Met were initially misdiagnosed with CIDP [31, 34]. Electrophysiological characteristics of ATTR-FAP can resemble those of CIDP; however, no symptoms of autonomic dysfunction are present [30, 31]. Cerebrospinal fluid protein levels are elevated to a greater extent than those seen in ATTR-FAP [30, 92]. Also, a nerve biopsy revealing congophilic deposit differentiates ATTR-FAP from CIDP [1]. ATTR-FAP should be suspected in patients diagnosed with CIDP that do not respond to immunomodulatory treatment if associated with steady progression of the neuropathy, an axonal pattern, and dysautonomia [30, 31, 46, 92].

#### AL amyloidosis

ATTR amyloidosis often was misdiagnosed as AL amyloidosis because of a high incidence of monoclonal gammopathy in elderly patients or false immunolabeling of amyloid deposits. However, this misdiagnosis can be avoided by careful typing of the amyloid precursor protein and genetic testing [1, 30, 91–93].

Other common misdiagnoses include idiopathic axonal polyneuropathy, other types of inherited sensory polyneuropathy, hereditary sensory and autonomic neuropathies, Fabry’s disease, leprous neuropathy, mimicking neuropathies due to diabetes or chronic alcoholism, Charcot–Marie–Tooth neuropathy or motor neuron disease, lumbar spinal stenosis, anxiety, and vitamin B12 deficiency [1, 18, 104].

### Management

The management of ATTR-FAP involves three primary steps [82]:

1. Disease-modifying targeted therapy to prevent further production of amyloid deposits (e.g. LT, transthyretin kinetic stabilizers such as tafamidis, diflunisal) [1, 35]
2. Symptomatic therapy of sensorimotor and autonomic polyneuropathy and cardiac, renal, and ocular injury [1, 35]
3. Genetic counseling and supportive care [1, 105]

**Fig. 5:**
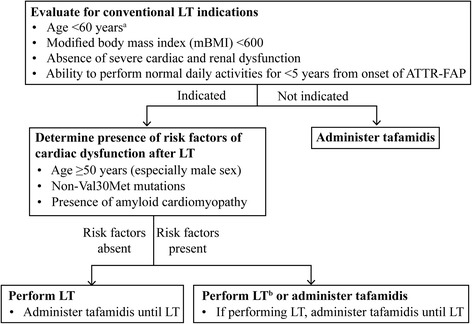
Treatment algorithm specific to ATTR-FAP Val30Met in Japan. ^a^Most late-onset ATTR-FAP patients show progression of the disease even after LT. However, our experience in Japan and evidence in literature suggest a good outcome after LT in some of the late-onset patients; especially, late-onset females showed significantly improved survival after LT than their male counterparts (*p* = 0.02, Okamoto S, et al. 2009 [108]; hazard ratio 1.57 [male vs female, *p* = 0.014], Ericzon BG, et al. 2015 [33]). ^b^Even in the late-onset cases, LT sometimes show good outcome in females (Ericzon BG, et al. 2015 [33]). The outcome of LT is comparably good in neuropathic type of non-Val30Met ATTR-FAP patients from our experience in Kumamoto University Hospital and Shinshu University Hospital in Japan (unpublished observations). *LT* liver transplantation, *ATTR-FAP* transthyretin familial amyloid neuropathy, *Val30Met* replacement of valine with methionine at position 30 in the TTR gene

Unlike European countries that have adopted pharmacotherapeutic treatment options for ATTR-FAP, LT remains first-line treatment in Japan [42, 106]. The Japan-specific ATTR-FAP treatment algorithm [107–109] developed at a medical advisory board meeting in Tokyo suggests that ATTR-FAP patients in Japan should initially be evaluated for presence of indications for LT. In patients who do not meet these indications, tafamidis should be administered. When indicated, patients should be further assessed for the presence of risk factors of cardiac dysfunction after LT. Patients without risk factors should undergo LT and be administered tafamidis until LT. Patients with risk factors may either undergo LT (tafamidis could be prescribed until transplantation) or may only be prescribed tafamidis (Fig. 5). Although ATTR-FAP disease duration <5 years is one of the indications for LT, some patients with mild symptoms, who meet other criteria may be considered for LT even if disease duration (from onset) is greater than 5 years [56, 110, 111].

#### LT

Since 1990, LT has been the only potentially curative and disease-modifying treatment option for ATTR-FAP patients [1, 36–41]. Serum TTR is mainly produced in the liver, and LT removes the primary source of mutant TTR, eliminates approximately 95% of variant TTR, and can slow or halt disease progression [1, 112–114]. A study that evaluated histopathological and biochemical characteristics of abdominal fat amyloid in patients who had undergone LT over 10 years earlier showed that tissue-deposited amyloid in FAP patients can gradually regress over the long term after LT [112]. Results from the Familial Amyloidotic Polyneuropathy World Transplant Registry (FAPWTR) initiated in 1995 show excellent patient survival (overall 5-year patient survival 77%, 20-year survival 55.3%), which is comparable to the survival rates seen in LT performed for other chronic liver disorders [33, 115]. The 20-year retrospective analysis by the FAPWTR also revealed that early disease onset, short disease duration, and the Val30Met mutation were significantly related to decreased mortality in LT patients (*p* < 0.001), while sex does not relate to increased survival for the early-onset LT patients (*p* = 0.442) [33]. A study of 80 consecutive patients with ATTR-FAP Val30Met who visited Kumamoto University hospital between January 1990 and December 2010 showed that Japanese patients undergoing LT have prolonged survival (*p* < 0.001) and higher (100% vs 56.1%) estimated probability of survival at 10 years after the onset of FAP [116]. In early-onset disease, significantly (*p* < 0.001) improved survival is observed in transplanted patients as compared to non-transplanted cases. However, in late-onset disease, survival of transplanted patients does not differ from that of non-transplanted patients [108]. Also, while early-onset cases showed no significant difference in survival after LT between male and female patients, late-onset disease female transplanted patients had significantly (*p* = 0.02) improved survival than male transplanted cases [108]. It is also noteworthy that 10-year survival rate after LT was numerically (but not significantly) better in patients who received a living-donor liver graft than those who received a graft from a deceased donor (72.3% vs 33.8%, *p* = 0.092) [117]. Another study of 45 patients with symptomatic ATTR-FAP showed overall 1- and 5-year survival rates of 82% and 60%, respectively, a marked reduction in circulating mutated TTR levels (2.5% of pre-LT values), and a markedly lower rate of axonal degeneration (0.9/mm^2^ vs 70/mm^2^ of endoneurial area/month in transplanted vs non-transplanted patients) after LT; LT at first symptom onset and exclusion of patients with a Norris score <55 and/or with urinary incontinence have been recommended [118]. Long-term survival after LT can be predicted by calculating the 5-year risk of death from the polyneuropathy disability (PND) score, presence or absence of orthostatic hypotension, New York Heart Association (NYHA) functional class, QRS duration, and interventricular septal thickness [119].

Of note, the situation surrounding the use of LT for ATTR-FAP in Japan is different from that in other areas of the world. Liver tissue from live donors is used for LT in Japan, whereas cadaveric liver tissue is used elsewhere [1]. Consequently, better LT treatment outcomes, including higher survival rates post LT, are achieved in Japan [116]. Therefore, despite the use of a recently approved therapy tafamidis, which is a first-line treatment option for patients with early-stage ATTR-FAP in Europe [42], LT remains the first-line treatment option in Japan, especially for early-onset ATTR-FAP Val30Met [106].

Despite being a standard therapeutic strategy for ATTR-FAP, LT has several limitations [113]. Organ impairment occurring before LT is not reversed [1]. As seen in the FAPWTR, the outcomes of LT are mutation-specific (10-year survival rate is 74% for Val30Met vs 44% for non-Val30Met patients; 20-year mortality rate in Val30Met patients is 61% that of non-Val30Met patients, *p* < 0.001) [1, 33]. Further, in some patients, disease progression occurs even after LT [62, 120]. For example, progression of cardiac amyloid infiltration continues post-LT because wild-type TTR continues to deposit on existing amyloid deposits [121–124]. Likewise, ocular and leptomeningeal deposits continue to increase after LT because of local, mutant TTR synthesis in the retinal epithelium and choroid plexus [61, 62, 113, 125–129]. Hence, although autonomic disturbances decrease post LT, nerve function rarely improves [1]. Also, in addition to the risks of surgery, long-term post-LT immunosuppressive therapy is required in these patients [1]. Further, many patients are not suitable candidates for LT, while in many others LT is not readily accessible [82, 130]. In addition, the risk of acquired systemic TTR amyloidosis in patients receiving domino LT should not be underestimated [131].

#### Pharmacotherapy

As destabilization of the TTR-tetramer along with misfolding and fibril formation contribute to its pro-amyloidogenic potential, TTR-tetramer stabilization was identified as a rate-limiting step and several new pharmacologic therapies such as TTR stabilizing agents were evaluated for the treatment of ATTR-FAP. These can be prescribed at an early stage of disease in anticipation of LT or to potentially delay the need for LT [1].

#### Tafamidis

Tafamidis (Vyndaqel®; Pfizer Inc.) approved in Europe in 2011 [42] and in Japan in 2013 is the only prescription drug for ATTR-FAP [132]. In addition to improved diagnostic techniques, availability of tafamidis prompted earlier diagnosis of cases from non-endemic areas, as it marked the transformation of ATTR-FAP from an uncontrollable condition into a treatable disease entity. Tafamidis, a disease-modifying agent, kinetically stabilizes mutant TTR tetramers and prevents their dissociation into monomers, which is a critical, rate-limiting step in fibril formation and amyloidogenesis [1, 133–135]. In a randomized, double-blind trial, where early-stage ATTR-FAP patients received tafamidis meglumine 20 mg (tafamidis 12.2 mg) once daily or placebo for 18 months, although no differences were observed between the tafamidis and placebo groups for the Neuropathy Impairment Score–Lower Limbs (NIS-LL) responder analysis (45.3% vs 29.5% responders; *p* = 0.068) and change in Norfolk Quality of Life Diabetic Neuropathy total score (TQOL; 2.0 vs 7.2; *p* = 0.116) in the intent-to-treat population (*n* = 125), a significantly greater proportion (60.0% vs 38.1%; *p* < 0.041) of patients receiving tafamidis were NIS-LL responders and tafamidis patients had better-preserved TQOL (0.1 vs 8.9; *p* = 0.045) in the efficacy-evaluable population (*n* = 87). Additionally, patients on tafamidis had better-preserved TQOL (0.1 vs 8.9; *p* < 0.045) and showed 52% less neurologic deterioration with adverse events (AEs) comparable to patients receiving placebo [32]. Another 12-month, open-label extension study that evaluated the long-term safety, tolerability, and efficacy of tafamidis 20 mg once daily in 86 patients showed reduced rates of neurological deterioration in patients treated with tafamidis for 30 months. Further, patients treated for 30 months had 55.9% greater preservation of neurologic function (as measured by the NIS-LL) than those in whom tafamidis was initiated later, thus demonstrating that early initiation of tafamidis was required to slow disease progression. Urinary tract infection, diarrhea, thermal burn, and nasopharyngitis were some of the most commonly observed AEs in the tafamidis group. However, no new safety or tolerability concerns were identified and the overall incidence of AEs and serious AEs was similar between tafamidis and placebo groups [42, 136]. Furthermore, an ongoing long-term, open-label extension study has revealed that early treatment with tafamidis for up to 5.5 years sustainably delayed neurologic progression and preserved nutritional status (mean changes from baseline: NIS-LL, 5.3 points; mBMI, −7.8 kg/m^2^ × g/L), without any new safety concerns [137].

In Japan, the efficacy and safety of tafamidis meglumine 20 mg (tafamidis 12.2 mg) once daily in ATTR-FAP patients (*n* = 10, male 70%, mean age 60.1 years) were evaluated for 1.5 years in a phase III, single-arm, open-label study [138]. The majority had the Val30Met mutation (90%) and were late-onset cases (70%, mean onset age 65.6 years). At week 8 of treatment, TTR stabilization was achieved in all the 10 patients (primary endpoint, percent stabilization ≥32%) and maintained over week 78 in 8 (80%) patients. Treatment with tafamidis delayed neuropathic progression (mean [SD] NIS-LL change at week 78, 3.3 [4.7]), maintained quality of life (mean [SD] TQOL change at week 78, 10.8 [13.7]) and improved nutritional status (mean [SD] mBMI increase at week 78, 53.7 [81.4]) over the study period. Nasopharyngitis, muscular weakness, bacterial pneumonia, and thermal burn were the most common AEs. Two AEs (gingival swelling and sudden death) in two patients were treatment-related, but no discontinuation due to AEs was observed [138]. These findings were consistent with previous tafamidis trials [32, 136] although generalizability is limited due to the small patient number and the non-comparative setting.

#### Diflunisal

Diflunisal, a generic nonsteroidal anti-inflammatory drug (NSAID), also slows the rate of amyloidogenesis by preventing the dissociation, misfolding, and misassembly of mutated TTR tetramers. Diflunisal preferentially stabilizes TTR tetramers by increasing the tetramer dissociation barrier via small molecule binding and by binding to the 99% unoccupied L-thyroxine binding sites in TTR [139, 140]. Because of high serum concentrations after oral administration, diflunisal imposes kinetic stability on TTR heterotetramers exceeding that of the wild-type homotetramer and compensates for its modest binding affinity and selectivity to TTR over all other serum proteins. Thus, diflunisal is the most promising NSAID for the treatment of TTR amyloidosis [140]. Diflunisal administered at a dose of 250 mg twice a day is sufficient to impose kinetic stabilization on the tetrameric native state of TTR and achieves kinetic stabilization under very demanding denaturing conditions. In an international randomized, double-blind, placebo-controlled study conducted among 130 ATTR-FAP patients in Sweden, Italy, Japan, England, and the United States from 2006 through 2012, polyneuropathy progression (measured by the Neuropathy Impairment Score plus 7 nerve tests [NIS+7]) was significantly less (NIS+7 score: 8.7 [95% confidence interval (CI), 3.3–14.1] vs 25.0 [95% CI, 18.4–31.6]) in patients receiving diflunisal. Also, patients on diflunisal showed significant improvement in quality of life measures than patients on placebo in whom quality of life deteriorated. Further, a greater proportion of patients receiving diflunisal (29.7% vs 9.4%) exhibited neurological stability at 2 years (<2-point increase in NIS+7 score; *p* = 0.007) [141]. A retrospective analysis of off-label use of diflunisal in patients with ATTR-FAP reported treatment discontinuation in 57% of patients due to gastrointestinal side effects [142].

The contraindication for NSAIDs in patients with severe congestive heart failure (NYHA class IV) or renal insufficiency (estimated creatinine clearance <30 mL/min) may limit its use in ATTR-FAP patients with cardiac or renal involvement [106, 141, 143]. Although the incidences of cardiac or renal events were similar in the diflunisal and placebo groups in a phase III study, two patients in the diflunisal group discontinued treatment due to gastrointestinal bleeding and congestive heart failure, respectively [141]. Because of risks of gastrointestinal bleeding, altered renal function, or fluid retention, patient selection, management of anti-inflammatory drug liabilities and long-term surveillance for AEs may be required [1, 139, 144].

#### Symptomatic therapy

The immediate goal of ATTR-FAP management is to alleviate symptoms; therefore, symptomatic management of sensory-motor neuropathy and autonomic dysfunction should be initiated immediately after diagnosis, irrespective of presenting symptoms [1]. Symptomatic treatments include prophylactic pacemaker implantation to reduce major cardiac events; medications to treat cardiomyopathy, pain, diarrhea, orthostatic hypotension, urinary incontinence, hypothyroidism, and cardiac failure; erythropoietin or iron for anemia; CTS-release surgery; hemodialysis for renal failure; and vitrectomy or trabeculectomy for ocular amyloidosis [1, 82, 145]. In a 45-month study, prophylactic pacemaker implantation mitigated major cardiac events in patients with polyneuropathy and conduction disorders [146]. Likewise, in older ATTR-FAP patients with cardiomyopathy, stabilization of fluid balance with a goal of reduction in filling pressure was achieved with very low doses of loop diuretics [106, 147].

#### Genetic counseling

Considering that genetic testing is a major tool for diagnosis and that it helps carrier detection in a genetic-counseling setting, relatives should be strongly encouraged to undergo genetic testing and tissue biopsies (in cases of TTR genopositivity) [1, 34]. However, as genetic testing in patients with a family history of ATTR-FAP may lead to severe anxiety, genetic counseling and psychological support for patients and their family members is necessary [1, 34]. Predictive genetic testing should be carried out in adult (aged ≥20) relatives of ATTR-FAP patients, once they are able to understand the medical, social, and psychological outcomes of a positive genetic test [1]. Also, during genetic counseling, individuals with a positive result should be made aware of the disease’s variable penetrance and the differences in age of symptom onset [24, 148, 149].

#### Supportive care

As management of ATTR-FAP is extremely challenging, it is important to provide patients and their families with all the social and moral support possible. Efforts should be intensified to achieve early identification and diagnosis. Symptomatic treatment should be initiated immediately, and a long-term strategy should be devised. The FAPWTR [150] was established for collaboration and exchange of experience, monitoring international transplant activity, and optimization of patient selection, to ensure satisfactory follow-up after transplant and to serve as an exploratory research tool for treatment centers. Other country- and region-specific networks and centers of excellence also should be established for exchange of clinical, pathological, and genetic evidence, and sharing of expertise and best management practices [82].

### Emerging therapies

A number of investigational pharmacologic treatments for ATTR-FAP are in development. Antisense oligonucleotide- and RNA interference-based therapeutics are two distinct methodologies aimed at reducing total TTR production [106]. ISIS-TTR_Rx_ is an antisense oligonucleotide-based therapy that causes destruction of wild type and mutant TTR transcripts. Within 12 weeks of treatment, ISIS-TTR_Rx_ treatment reduced hepatic TTR mRNA and serum TTR protein levels by 80% in mouse and non-human primate models [151, 152]. Patisiran (ALN-TTR02) employs TTR-targeting, small interfering RNAs to reduce wild-type and mutant TTR. In a phase II, open-label, multidose, dose-escalation study involving 29 patients with stage I or II h-ATTRm amyloidosis with polyneuropathy, patisiran 0.3 mg/kg every 3 weeks resulted in a maximum mean reduction of 87% in TTR level; a maximum of 96% was attained in one patient [153]. Likewise, revusiran (ALN-TTRsc), a subcutaneously administered and TTR-targeting siRNA conjugated to a triantennary, reduced TTR protein expression by approximately 80% in non-human primate models at doses as low as 2.5 mg/kg [154].

Synergistic combination of doxycycline (an in vitro TTR fibril disrupter) and tauroursodeoxycholic acid (TUDCA; a biliary acid acting as a potent anti-apoptotic and anti-oxidant) has demonstrated removal of amyloid deposits in mouse models [155], and clinical trials (NCT01855360, NCT01171859) are seeking to replicate these findings in patients. Finally, endogenous and exogenous monoclonal antibodies can be used to target amyloid deposits [106, 156]. A humanized, anti-amyloid, monoclonal antibody NEOD001 and the combination of a serum amyloid P depleter (GSK2315698) and an anti-serum amyloid P monoclonal antibody (GSK2398852) are being tested in patients with various forms of amyloidosis (NCT01707264, NCT01777243).

## Conclusions

Phenotypic and genetic heterogeneity may delay diagnosis of ATTR-FAP in Japan. The Japan-specific red-flag symptom clusters proposed herein may simplify diagnosis for physicians and prevent misdiagnosis or delayed diagnosis of ATTR-FAP. Likewise, our consensus-based ATTR-FAP treatment algorithm, which was also based on treatment outcomes observed in Japan, may guide clinicians regarding apt and judicious use of available treatment modalities.

## Abbreviations

AE: Adverse event
ATTR: Transthyretin
ATTRwt: Wild-type transthyretin
CI: Confidence interval
CIDP: Chronic inflammatory demyelinating polyneuropathy
CTS: Carpal tunnel syndrome
FAP: Familial amyloid polyneuropathy
FAPWTR: Familial Amyloidotic Polyneuropathy World Transplant Registry
h-ATTRm: hereditary transthyretin
LT: Liver transplantation
mBMI: modified body mass index
MHLW: Ministry of Health, Labour and Welfare, Japan
NIS+7: Neuropathy Impairment Score plus 7 nerve tests
NIS-LL: Neuropathy Impairment Score–Lower Limbs
NSAID: nonsteroidal anti-inflammatory drug
NYHA: New York Heart Association
PND: Polyneuropathy disability
TQOL: Norfolk Quality of Life Diabetic Neuropathy total score
TTR: Transthyretin
TUDCA: Tauroursodeoxycholic acid
Val30Met: Replacement of valine with methionine at position 30
