# Correction to: Diagnosis and management of transthyretin familial amyloid polyneuropathy in Japan: red-flag symptom clusters and treatment algorithm

**DOI:** 10.1186/s13023-019-1092-7

**Published:** 2019-05-21

**Authors:** Yoshiki Sekijima, Mitsuharu Ueda, Haruki Koike, Sonoko Misawa, Tomonori Ishii, Yukio Ando

**Affiliations:** 1 Department of Medicine (Neurology and Rheumatology), Shinshu University School of Medicine, Matsumoto, Japan; 2 Department of Neurology, Graduate School of Medical Sciences, Kumamoto University, 1-1-1 Honjo, Chuo-ku, Kumamoto-shi, Kumamoto, 860-8556 Japan; 3 Department of Neurology, Nagoya University Graduate School of Medicine, Nagoya, Japan; 4 Department of Neurology, Graduate School of Medicine, Chiba University, Chiba, Japan; 5 Pfizer Japan Inc., Tokyo, Japan

**Correction to: Orphanet Journal of Rare Diseases (2018) 13:6**

**https://doi.org/10.1186/s13023-017-0726-x**

The authors would like to rectify the following errors in their published article [1]:

In Fig. 3 of the article (page 7 of the PDF), *‘Val30Met’* should be omitted from the figure legend, which should instead read: *‘Red-flag symptom clusters specific to ATTR-FAP in Japan…’.*

Similarly, in Fig. 5 of the article (page 10 of the PDF), *‘Val30Met’* should be omitted from the figure legend, which should instead read: *‘Treatment algorithm specific to ATTR-FAP in Japan…’.*

The authors believe that the applicability of the red-flag symptom clusters and treatment algorithm for this disease should not be limited to the Val30Met mutation and would like to present this with this Correction article.

Furthermore, in Fig. 5 of the article (page 10 of the PDF), the treatment algorithm, *‘Modified body mass index (mBMI) <600’*, listed as the 2nd bullet under ‘Evaluate for conventional LT indications’, should be changed to *‘Modified body mass index (mBMI) >600’*.

This error occurred due to an inadvertent oversight.
